# The Impact of Down Syndrome Screening on Taiwanese Down Syndrome Births: A Nationwide Retrospective Study and a Screening Result from a Single Medical Centre

**DOI:** 10.1371/journal.pone.0075428

**Published:** 2013-09-20

**Authors:** Shin-Yu Lin, Chia-Jung Hsieh, Yi-Li Chen, Sheng-Wen Steven Shaw, Ming-Wei Lin, Pau-Chung Chen, Chien-Nan Lee

**Affiliations:** 1 Department of Obstetrics and Gynecology, National Taiwan University Hospital, Hsin Chu Branch, Hsin Chu, Taiwan; 2 Graduate Institute of Clinical Medicine, National Taiwan University College of Medicine, Taipei, Taiwan; 3 Department of Public Health, Tzu-Chi University, Hualien, Taiwan; 4 Graduate Institute of Molecular Medicine, National Taiwan University College of Medicine, Taipei, Taiwan; 5 Department of Obstetrics and Gynecology, Chang Gung Memorial Hospital, Linkou Medical Centre and Chang Gung University College of Medicine, Taoyuan, Taiwan; 6 Department of Obstetrics and Gynecology, National Taiwan University Hospital, Taipei, Taiwan; 7 Institute of Occupational Medicine and Industrial Hygiene, National Taiwan University College of Public Health, Taipei, Taiwan; IGBMC/ICS, France

## Abstract

A retrospective analysis of the Taiwanese National Birth Defect Registration and Notification System was conducted in order to determine the live birth- and stillbirth rates in infants with Down syndrome, trisomy 18, trisomy 13 and Turner syndrome between 2001 and 2010. The objective was to investigate the impact of Down syndrome screening on the Taiwanese Down syndrome live birth rate. In addition, the results of first-trimester Down syndrome screening between 2006 and 2011, and of second-trimester quadruple testing between 2008 and 2011, were obtained from the National Taiwan University Hospital. All Taiwanese infants born between 2001 and 2010 were included in the first part of the analysis, and women receiving first-trimester Down syndrome screening or second-trimester quadruple testing from the National Taiwan University Hospital were included in the second part. The live birth rate of infants with Down syndrome, per 100 000 live births, decreased from 22.28 in 2001 to 7.79 in 2010. The ratio of liveborn DS to total DS was 48.74% in 2001, and then decreased to 25.88% in 2006, when first-trimester screening was widely introduced in Taiwan. This ratio dropped to 20.64% in 2008, when the second-trimester quadruple test was implemented. The overall positive rate in first-trimester screening in the National Taiwan University Hospital was 3.1%, with a Down syndrome detection rate of 100%; the quadruple test had values of 9.0% and 75%, respectively. The use of first-trimester screening and the second-trimester quadruple test may be responsible for the marked decrease in the Taiwanese Down syndrome live birth rate observed between 2001 and 2010.

## Introduction

Antenatal Down syndrome (DS) screening has become widely available around the world and it is recommended that an evaluation for aneuploidy, either a screening test or a more invasive, definitive test, should be offered to all pregnant women [1]. There are many strategies available to screen for DS, incorporating maternal age and a variety of first- and second-trimester ultrasound and biochemical markers. First-trimester DS screening involves the ultrasound measurement of fetal nuchal translucency (NT) thickness, typically increased in fetuses with DS [1], combined with the assessment of maternal concentrations of serum free beta-human chorionic gonadotrophin (β-hCG) and pregnancy-associated plasma protein-A. Second-trimester screening involves the maternal measurement of serum β-hCG, alpha-fetoprotein (AFP) and unconjugated estriol concentrations to make up the triple screen, with the addition of inhibin A when quadruple screening is desired. A double screen may also be performed, with the assessment of β-hCG and AFP concentrations [2,3,4,5,6]. All of these approaches provide the mother with an age-adjusted risk for DS, as the concentrations of AFP, pregnancy-associated plasma protein-A and unconjugated estriol are known to be decreased when fetuses are affected with DS, while β-hCG and inhibin A are increased [1]. Over the past 20 years, advances in testing have resulted in a larger proportion of affected pregnancies being detected with fewer women requiring invasive diagnostic testing.

According to the current public health policy in Taiwan, amniocentesis is indicated in pregnant women aged 35 years and over, although it is suggested that advanced maternal age is no longer the sole determinant of whether a patient is offered screening versus invasive testing [1,7]. The second-trimester double test was introduced in Taiwan in 1994, and the ratio of DS live birth to total DS subsequently dropped significantly, from 70.42% in 1994 to 48.74% in 2001 [8]. First-trimester DS screening was implemented in Taiwan in 2001, and second-trimester quadruple screening was added in 2008.

We used birth certificate data to investigate the trend in live births, between 2001 and 2010, for Taiwanese infants with DS. We also analyzed the DS screening results at a single medical centre between 2006 and 2011, in order to illustrate any effect of the first-trimester DS screening program and/or the second-trimester quadruple test. We found that use of this testing may be responsible for the marked decrease in the DS live birth rate in Taiwan observed between 2001 and 2010.

## Methods

This study was approved by the Research Ethics Committee of National Taiwan University Hospital (201210077RIC). Data from the Taiwanese National Birth Defect Registration and Notification System and from the Bureau of health promotion, department of health, Taiwan were accessed; births taking place between 2001 and 2010 were included for analysis. This national database was generated from standard registration forms filled out by clinicians at hospitals and clinics in Taiwan for each newborn, reporting birth defects, infants with abnormal karyotypes (when known) and abnormalities detected during the perinatal period. The annual number of live births were obtained from Department of statistics, Ministry of the Interior, executive yuan, Taiwan.

DS infants were reported as live births or stillbirths, without regard to gestational age. Stillbirths included both intrauterine demises and pregnancy terminations after the prenatal diagnosis of DS. The amniocentesis rate was obtained from the Bureau of health promotion, department of health, Taiwan, which was started to be registered since 2006. The number of women receiving first-trimester screening was taken from the number of tests submitted to the Fetal Medicine Foundation (http://www.fetalmedicine.com/fmf/). However, the individual outcomes for pregnancies tested by either first-trimester DS screening or second-trimester quadruple testing were not available.

We collected the first-trimester DS screening and second-trimester quadruple testing results for the period between 2006 and 2011 from the National Taiwan University Hospital (NTUH), a tertiary medical centre in Taiwan with around 2500 to 3000 deliveries each year. The second-trimester DS double test was used at NTUH between 1994 and 2007; in 2006, the first-trimester DS screening program was implemented, with the second-trimester quadruple test replaced the second-trimester DS double test at NTUH in 2008. During the study period, there were 8 sonographers at NTUH certified by the Fetal Medicine Foundation to perform NT assessment.

According to national policy, most Taiwanese pregnant women aged 35 years or greater would have been offered amniocentesis during the study period. When NTUH adopted first-trimester DS screening, a contingent program was put in place. Women with a screening risk of 1 in 270 or greater were considered to be at high risk of having a fetus with DS and were referred for amniocentesis. Women with a risk between 1 in 270 and 1 in 1000 were considered to have an intermediate risk of having a fetus with DS and the second-trimester quadruple test was recommended (contingent sequential screening [1]). Women with a risk of less than 1 in 1000 received regular prenatal care. On second-trimester quadruple testing, women with a screening risk of 1 in 270 or greater were considered to be at high risk of having a fetus with DS and were referred for amniocentesis. There were unfortunately no data on how many women over the age of 35 received amniocentesis primarily versus how many underwent screening, then amniocentesis.

## Results

**Figure 1:**
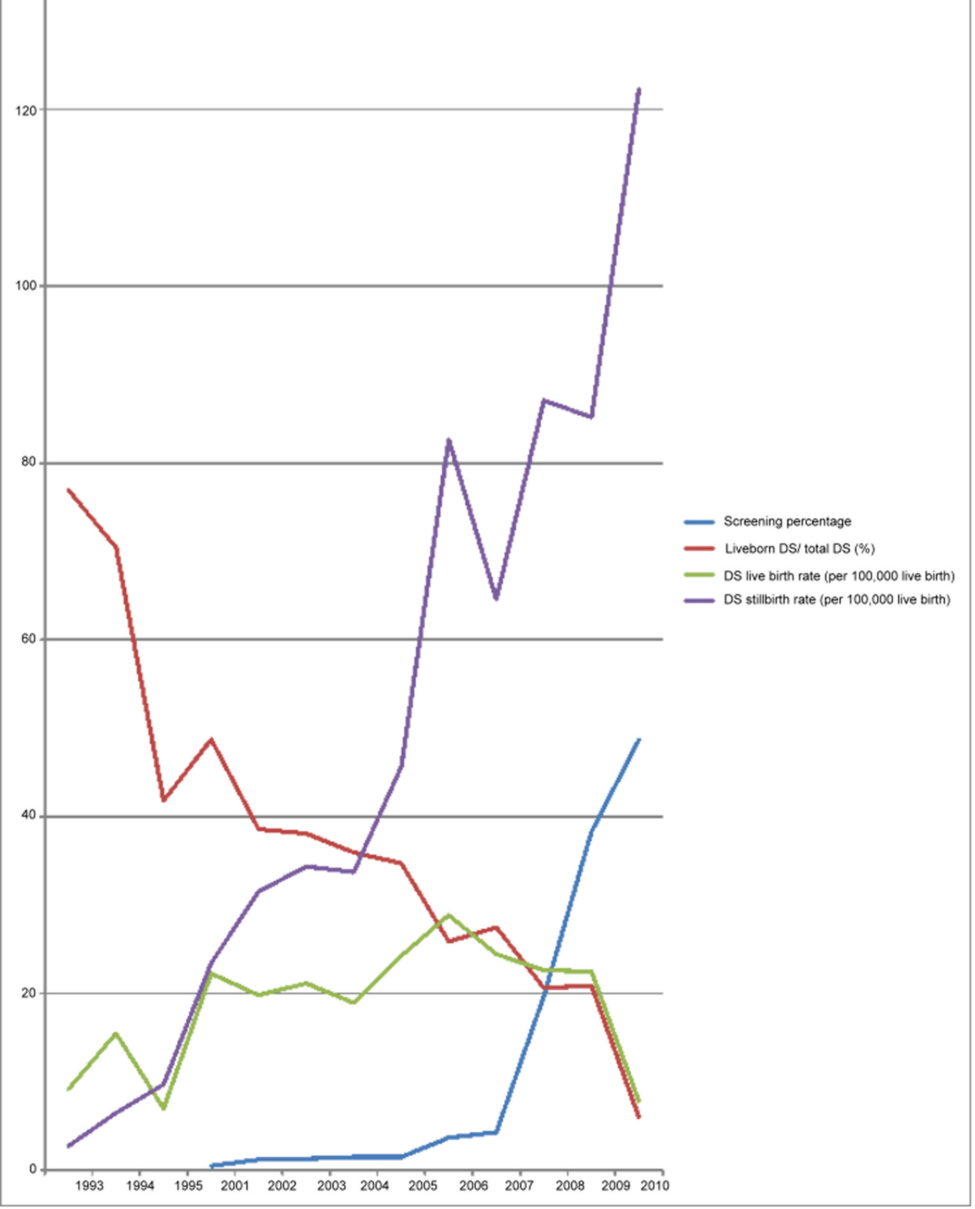
Down syndrome (DS) live birth rates. The Taiwanese DS live birth ratio dropped from 48.74% of all fetuses with DS in 2001 to 25.88% in 2006, when first-trimester DS screening was widely introduced. The rate dropped further, to 20.64%, in 2008, when the second-trimester quadruple test was implemented in Taiwan. By 2010, the live birth rate was 7.79 per 100 000 live births.

The records of 2 152 228 deliveries were collected between 2001 and 2010 in Taiwan; these deliveries included 2 123 029 live births, 29 199 stillbirths and 1681 infants with DS confirmed by karyotype analysis (Table 1). The numbers of pregnant women over 35 years of age increased each year, triple from 1993 to 2010. The incidence of DS was 7.92 per 10 000 live births between 2001 to 2010; that is, one out of every 1263 live births resulted in an infant with DS. The Taiwanese DS live birth ratio dropped from 48.74% of all fetuses with DS in 2001 to 25.88% in 2006, when first-trimester DS screening was widely introduced. The rate dropped further, to 20.64%, in 2008, when the second-trimester quadruple test was implemented in Taiwan (Cochran-Armitage Trend Test, P<0.0001). By 2010, the live birth rate was 7.79 per 100 000 live births. Nearly half of all pregnant women in Taiwan received either the first-trimester screen or the second-trimester quadruple test in 2010 (Table 1); over 95% of women underwent either the DS maternal serum screening or amniocentesis. The invasive prenatal diagnosis uptaken rate rose from 12.22% in 2006 to 20.11% in 2010. The percentage of women aged over 35 year-old and received invasive prenatal diagnosis also increased from 63.28% in 2006 to 75.35% in 2010. We did not have data on the number of women that underwent the double-test. The overall trend of DS birth rates is demonstrated in Figure 1. The years 1994 to 1995 demonstrated a crossover, from more DS live births to more DS stillbirths, coinciding with the implementation of the second-trimester double test in 1994 [8]. The ratio of DS to total DS was decreasing, and the stillbirth rate was significantly increasing (both p<0.0001), parallel to the DS serum screening uptaken rate (p<0.0001). However, the DS live birth rate per 100 000 live births was not statistically significantly different form 2001 to 2010, which might be influenced by the decreasing total live birth number. But it is also shown that the DS live birth rate per 100 000 live births was reduced since 2006, when DS serum screening became more and more available.

**Table 1:** 

	1993^&^	1994^&^	1995^&^	2001	2002	2003	2004	2005	2006	2007	2008	2009	2010
Registered live births^#^	325994	322938	329581	260,354	247,530	227,070	216,419	205,854	204,459	204,414	198,733	191,310	166,886
Maternal age ≧ 35 years	16887 (5.18%)	18112 (5.61%)	20003 (6.07%)	23046 (8.85%)	21851 (8.83%)	21468 (9.45%)	21472 (9.92%)	22635 (11.70%)	23920 (11.70%)	25260 (12.36%)	26389 (13.28%)	27683 (14.47%)	28689 (17.19%)
Invasive prenatal diagnosis									24985 (12.22%)	26486 (12.96%)	27645 (13.91%)	31939 (16.69%)	33553 (20.11%)
Maternal age ≧ 35 years									15,137	16,957	17,582	20,226	21,617
(Percentage)									63.28%	67.13%	66.63%	73.06%	75.35%
Maternal age < 35 years									9,848	9,529	10,063	11,713	11,936
(Percentage)									5.45%	5.32%	5.84%	7.16%	8.64%
Registered live DS births	30	50	23	58	49	48	41	50	59	50	45	43	13
Registered DS stillbirths	9	21	32	61	78	78	73	94	169	132	173	163	204
Incidence of DS (per 100,000 live birth)	12	22	17	46	51	55	53	70	112	89	110	108	130
Liveborn DS/ total DS^%^	76.92%	70.42%	41.82%	48.74%	38.58%	38.10%	35.96%	34.72%	25.88%	27.47%	20.64%	20.87%	5.99%
DS live birth rate (per 100,000 live birth) ^$^	9.20	15.48	6.98	22.28	19.80	21.14	18.94	24.29	28.86	24.46	22.64	22.48	7.79
DS stillbirth rate (per 100,000 live birth) ^$ $^	2.76	6.50	9.71	23.43	31.51	34.35	33.73	45.66	82.66	64.57	87.05	85.20	122.24
Registered live T18 births				3	3	5	5	7	7	2	3	5	1
Registered T18 stillbirths				15	21	30	42	27	35	29	26	31	27
Incidence of T18				0.73%	0.99%	1.55%	2.16%	1.64%	2.05%	1.52%	1.48%	1.87%	1.68%
Liveborn T18/ total T18				16.67%	12.50%	14.29%	10.64%	20.59%	16.67%	6.45%	10.34%	13.89%	3.57%
T18 live birth rate^*^				1.21	1.24	2.22	2.3	3.38	3.41	0.98	1.53	2.6	0.6
T18 stillbirth rate^*^				6.07	8.69	13.32	19.32	13.05	17.07	14.26	13.24	16.11	16.2
Registered live T13 births				2	2	1	0	0	0	5	0	1	0
Registered T13 stillbirths				8	5	3	10	6	9	3	13	6	7
Incidence of T13				0.40%	0.29%	0.18%	0.46%	0.29%	0.44%	0.39%	0.66%	0.36%	0.42%
Liveborn T13/ total T13				20.00%	28.57%	25.00%	0	0	0	62.50%	0	14.29%	0
T13 live birth rate^*^				0.81	0.83	0.44	0	0	0	2.46	0	0.52	0
T13 stillbirth rate^*^				3.24	2.07	1.33	4.6	2.9	4.39	1.48	6.62	3.12	4.2
Registered TS live births				0	2	3	2	3	3	1	2	1	1
Registered TS stillbirths				0	9	19	12	12	9	10	12	10	8
Incidence of TS				0	0.46%	0.98%	0.64%	0.72%	0.59%	0.54%	0.71%	0.57%	0.54%
Liveborn TS/ total TS				NA	0.18	0.14	0.14	0.2	0.25	0.09	0.14	0.09	0.11
TS live birth rate^*^				0	0.83	1.33	0.92	1.45	1.46	0.49	1.02	0.52	0.6
TS stillbirth rate^*^				0	3.72	8.43	5.52	5.8	4.39	4.92	6.11	5.2	4.8
Women receiving 1st trimester Down screening				1367	3045	3038	3229	3210	7643	8921	11320	12443	14173
Women receiving 2nd trimester quadruple Down screening											27723	62136	67964
Screening percentage				0.50%	1.20%	1.30%	1.50%	1.50%	3.70%	4.30%	19.70%	38.30%	48.70%

^#^ Data from the Department of statistics, Ministry of the Interior, executive yuan, Taiwan.

^%^ Cochran-Armitage Trend Test, P<0.0001

^$^ Poisson regression for trend, p=0.32

^$$^ Poisson regression for trend, p<0.0001

A total of 4106 women at NTUH received first-trimester DS screening between 2006 and 2010; their pregnancies comprised 3946 singletons, 158 sets of twins and 2 sets of triplets. Of these women, 3236 (78.8%) were 34 years of age or younger. High-risk results were noted in 107 singleton pregnancies and 20 twin pregnancies. Of these 127 women, 115 (90.6%) received invasive testing to obtain a karyotype; 9 underwent chorionic villus sampling and 106 underwent amniocentesis. The overall positive rate for first-trimester DS screening was 3.1%, with a DS detection rate of 100%.

First-trimester screening did not detect only DS: fetuses with trisomy 18, trisomy 13, Turner syndrome, the 6p25 microdeletion syndrome, skeletal dysplasia and intrauterine fetal demise were also found (Table 2). Of the 211 patients determined to be at intermediate risk, a few abnormal pregnancies were identified: 1 fetus had triploidy, 1 had mosaic DS, 1 had a Yq12 deletion and 1 had renal agenesis.

**Table 2:** 

	Total	First trimester screen Down syndrome risk	Second trimester quadruple test Down syndrome risk	Maternal age	Nuchal translucency (mm)	Karyotype	Remark
First trimester Down syndrome screening (9/2006-12/2011)	4106						
High risk	127						Screening positive rate: 3.1%; Detection rate: 100%
		1/2		32	6	Trisomy 18	Terminated
		1/2		30	6.4	Trisomy 18	Terminated
		1/143		42	9.5	Trisomy 18	Terminated
		1/2		32	6	Trisomy 21	Terminated
		1/2		34	1.3	Trisomy 21	Terminated
		1/2		33	7.6	45,X	Cystic hygroma, terminated
		1/91		33	1.5	45,X	Terminated
		1/22		44	7.5	Trisomy 13	Megacystic, SUA, tachycardia, terminated
		1/111		33	3.6/3.1	both 46, XY	Twins, one IUFD at the 17th week of gestation
		1/2; 1/292		29	8.9/2.0	both 46, XY	Twins, one IUFD at the 15th week of gestation, hydrops fetalis
		1/102		35	3.5	6p25 microdeletion	Severe TR, AC, complex CHD, termination at the 17th week of gestation
		1/19		33	2	46, XX	Oligohydromnios and IUFD at the 23rd week of gestation
		1/47		30	2.8	46, XY	Thanotophoric dysplasia, terminated
		1/4		43	1.4	46, XY	Skeletal dysplasia, terminated at the 32nd week of gestation
Intermediate risk	211						
		1/412		35	1.1	69,XXX	Immature fetus delivered spontaneously at 22 weeks; birth weight 170 g
		1/973		29	2.8	mos 47,XY,+21 [7]/46,XY[5]	Terminated
		1/305		29	3	46,xy	IUFD at the 22nd week of gestation
		1/828	1/5030	32	1.9	46,xy	Hydrops fetalis,terminated
		1/310	<1/38500	33	2.6		IUFD at the 23rd week of gestation
		1/749		29	2.3	46,X, del(Y) (q12)dn	Live birth
		1/672		32	1.7	45, X	Terminated
		1/302	1/61	30	1.8	46, XX	Live birth
Second trimester quadruple Down syndrome screening (5/2008-12/2011)	1307						Screening positive rate: 9.0%; Detection rate: 75%
High risk	118						
			1/122	33		47, XX,+21	Terminated
			1/6	31		47, XY,+21	Terminated
			1/6	41		47, XY,+21	Terminated
			1/1110	41		47, XY,+21	Pregnancy at 18th week of gestation with multiple fetal anomalies, terminated

The second-trimester double test was abandoned since 2008 in NTUH. Of the 1307 women receiving the second-trimester quadruple test at NTUH, 1060 (81.1%) were under 35 years of age. Four fetuses were determined to have DS, 1 of which had a screening risk of one in 1110 and was identified due to multiple structural abnormalities seen on ultrasound. The overall positive rate of the quadruple test for DS was 9.0%, with a DS detection rate of 75%.

## Discussion

DS is the most common chromosomal abnormality in infants, and one of the most common causes of mental retardation and congenital malformations. Prior to 2001, the reported incidence of DS in Taiwan was one in 848 live births, or 11.8 per 10 000 live births [9], although the poor registration system seems to have led to an overestimation of this incidence. With an improved registration system in place beginning in 2001, the incidence of DS between 2001 and 2010 has been determined to be one in 1263 live births, or 7.9 per 10 000 live births. This incidence is close to that reported in Japan (8.3-9.7 per 10 000 live birth) but lower than that seen in Europe (22.0 per 10 000 live birth) (Table 3) [10,11]. In the United States, the incidence of DS itself is reported to have been stable, in the range of 9.8 to 11.8 per 10 000 live births between 1985 and 1993 [12]. Reported prevalence of Down syndrome may be affected by the maternal age distribution of the population, early fetal loss due to trisomy 21, the rate of cytologic confirmation of fetal demise, prenatal testing availability and accuracy of diagnosis. Around 5% of spontaneous abortion before the 25^th^ week of gestation is resulted from trisomy 21 [13,14,15]. Nevertheless, there are no data on very early fetal losses due to DS in this study. Since 2001, the National Birth Defect Registration and Notification System has registered over 97% of all births in Taiwan. The DS live birth rate markedly decreased after Down syndrome screening was introduced to Taiwan and public health policy called for mothers 35 years of age or greater to be routinely referred for amniocentesis. The rate of invasive prenatal diagnosis increased from 12.22% in 2006 to 20.11% in 2010 (Table 1). Over 95% of women during the study period were evaluated by either screening tests or amniocentesis.

**Table 3:** Incidence of Down syndrome.

Country	Years	DS incidence (per 10,000 live births)	Reference
Canada	1993-1997	12.79	[26]
United States	1985-1993	9.8-11.8	[27]
Brazil	1993-1998	15.01	[26]
Norway	1993-1998	10.28	[26]
Finland	1993-1998	11.75	[26]
England & Wales	1993-1998	5.38	[26]
Europe	1990-2009	22.02	[11]
France Paris	1981-2000	25.9	[28]
Australia	1993-1997	13.14	[26]
New Zealand	1994-1998	9.9	[26]
United Arab Emirates	1996-1998	17.65	[26]
Japan	1980-1997	5.82 (8.3-9.7*)	[10]
Singapore	1993-1998	10.2	[29]
Taiwan	2001-2010	7.9	This study

* After correction according to the estimated ascertainment ratio: 60–70%.

Combining the data from previous literature and the present study, we demonstrated that the ratio of DS live birth to total DS in Taiwan has markedly decreased from 70.42% in 1994 to 41.82% in 1995 [8], then to 25.88% in 2006 and 5.99% in 2010. The lowest DS live birth rate was observed in 2010, at 7.79 per 100 000 live births. This change may be attributable to the introduction and the liberal use of the second-trimester double test, first-trimester DS screening and the second-trimester quadruple test in 1994, 2006 and 2008, respectively and increasing use of amniocentesis as well.

The second-trimester double test, still used in about half of Taiwanese pregnancies, takes into account maternal age, AFP and β-hCG. It was not included in our analysis of test results, although 2 Taiwanese hospital-based reports demonstrated a detection rate of 59.5–62.5% for the double test, with a false-positive rate of 5.9–6.5%; these results are comparable to those seen in Western countries [8,16,17]. Only 1 tertiary center in Taiwan has adopted the triple test, with unconjugated estriol as the third marker. One study reported the detection rate of the triple test for DS in a Taiwanese population to be 78.6% [18], although ACOG quotes a rate of only 69% [1]. The DS detection rate of the second-trimester quadruple test in Taiwan is 81.8%, with a 4.4% false-positive rate, comparable to the rates seen in Western countries [1,19]. According to guidelines published by the American Congress of Obstetricians and Gynecologists (ACOG), either serum integrated testing or second-trimester triple or quadruple testing should be offered to pregnant women when certificated practitioners are not available to perform first-trimester combined testing with NT measurement [1]. In terms of detection rate, cost-effectiveness and safety (i.e. avoiding unnecessary invasive testing), the NT measurement, the quadruple test, the first-trimester combined screen and the integrated tests (using both first- and second-trimester testing to reach a final result, with or without NT testing [1]) represent the best options for DS screening. These tests are preferable to screening based only on maternal age and either the second-trimester double test or the first-trimester serum test [3,20].

Although maternal DS screening also provides the risk of carrying a fetus with trisomy 18 or trisomy 13, the live birth rate for infants with these disorders has not decreased with the introduction of maternal DS screening. This may be partly due to the low incidence of trisomy 18 and trisomy 13, and partly due to the lower detection rates for these disorders, compared with the detection rate for DS, on maternal screening performed using the algorithm for DS detection (maternal age, serum testing and NT measurement) [21]. In addition, most fetuses with trisomy 18 and trisomy 13 have multiple anomalies, and detection therefore is mainly accomplished by ultrasound and amniocentesis. A similar situation applies in Turner syndrome: although 100% of fetuses with Turner syndrome are detected by the first-trimester DS screen [22], the early onset of cystic hygroma and the high rate of intrauterine demise may lead to pregnancy termination (spontaneous or voluntary) without maternal DS screening or karyotype analysis [22].

ACOG recommends that all women, regardless of their age, be offered aneuploidy screening before they have completed 20 weeks of gestation. Their guidelines also state that all women should have access to invasive diagnostic testing, should they desire this option [1]. DS screening programs are not aimed at eliminating children with DS, but rather at the early diagnosis of the condition. There are multiple screening approaches available, and it is important to keep in mind that all patients may not have access to NT measurement. Screening based on second-trimester markers alone continues to be an option for these patients, as well as for those who present to care too late for first-trimester testing [23]. Earlier testing is preferable, however, because early ultrasound dating reduces the risk of false-positive screening tests, as serum markers can be accurately matched to the gestational age [23].

With advances in molecular technology, noninvasive methods are now available for the prenatal diagnosis of chromosomal and other abnormalities. In particular, the isolation and examination of fetal DNA in the maternal circulation is now possible. Two large-scale independent studies have validated this testing technique; the studies respectively demonstrated a DS detection rate of 98.6% and 100%, with false-positive rates of 0.2% and 1.1% [24,25]. This testing modality is expensive, however, and requires sophisticated sequencing protocols and bioinformatics algorithms.

The limitations of the present study include the fact that the nationwide DS screening results and information on fetal outcomes were not available. Underreporting may have occurred, affecting the observed results. The overall trends that we report may be influenced by many factors other than screening-test results, including rising maternal age and the increasing availability of amniocentesis. Antenatal DS serum screening is known to be less sensitive in multiple gestations. The DS live birth rate given in this study may not completely represent the phenomenon of DS misdiagnosis.

## Conclusions

We have demonstrated that the liberal use of first-trimester DS screening and the second-trimester quadruple test may be responsible for the marked decrease in the ratio of liveborn DS to total DS in Taiwan observed between 2001 and 2010. Around half of the women in Taiwan are still using the second-trimester double test as their DS screening tool. Replacing this test with other effective screening programs and adopting the noninvasive fetal trisomy test is a worthy goal.

## References

[1] ACOG (2007) Screening for fetal chromosomal abnormalities. Obstet Gynecol 109: 217-227. doi:10.1097/00006250-200701000-00054. PubMed: 17197615. 17197615

[2] Spencer K, Spencer CE, Power M, Dawson C, Nicolaides KH (2003) Screening for chromosomal abnormalities in the first trimester using ultrasound and maternal serum biochemistry in a one-stop clinic: a review of three years prospective experience. BJOG 110: 281-286. doi:10.1046/j.1471-0528.2003.02246.x. PubMed: 12628268. 12628268

[3] Wald NJ, Huttly WJ, Hackshaw AK (2003) Antenatal screening for Down’s syndrome with the quadruple test. Lancet 361: 835-836. doi:10.1016/S0140-6736(03)12680-3. PubMed: 12642052. 12642052

[4] Benn PA, Fang M, Egan JF, Horne D, Collins R (2003) Incorporation of inhibin-A in second-trimester screening for Down syndrome. Obstet Gynecol 101: 451-454. doi:10.1016/S0029-7844(02)03159-9. PubMed: 12636947. 12636947

[5] Kellner LH, Weiner Z, Weiss RR, Neuer M, Martin GM et al. (1995) Triple marker (alpha-fetoprotein, unconjugated estriol, human chorionic gonadotropin) versus alpha-fetoprotein plus free-beta subunit in second-trimester maternal serum screening for fetal Down syndrome: a prospective comparison study. Am J Obstet Gynecol 173: 1306-1309. doi:10.1016/0002-9378(95)91376-9. PubMed: 7485343. 7485343

[6] Wald NJ, Cuckle HS, Densem JW, Nanchahal K, Royston P et al. (1988) Maternal serum screening for Down’s syndrome in early pregnancy. BMJ 297: 883-887. doi:10.1136/bmj.297.6653.883. PubMed: 2460174. 2460174 PMC1834444

[7] Haddow JE, Palomaki GE, Knight GJ, Cunningham GC, Lustig LS et al. (1994) Reducing the need for amniocentesis in women 35 years of age or older with serum markers for screening. N Engl J Med 330: 1114-1118. doi:10.1056/NEJM199404213301603. PubMed: 7510852. 7510852

[8] Jou HJ, Kuo YS, Hsu JJ, Shyu MK, Hsieh TT et al. (2005) The evolving national birth prevalence of Down syndrome in Taiwan. A study on the impact of second-trimester maternal serum screening. Prenat Diagn 25: 665-670. doi:10.1002/pd.1220. PubMed: 16049991. 16049991

[9] Lin S (1991) Incidence and growth of Down syndrome in Taiwan. Scientific Study Report of Department of Health, Republic of China.

[10] Hoshi N, Hattori R, Hanatani K, Okuyama K, Yamada H et al. (1999) Recent trends in the prevalence of Down syndrome in Japan, 1980-1997. Am J Med Genet 84: 340-345. doi:10.1002/(SICI)1096-8628(19990604)84:4. PubMed: 10340648. 10340648

[11] Loane M, Morris JK, Addor MC, Arriola L, Budd J et al. (2013) Twenty-year trends in the prevalence of Down syndrome and other trisomies in Europe: impact of maternal age and prenatal screening. Eur J Hum Genet 21: 27-33. doi:10.1038/ejhg.2012.94. PubMed: 22713804. 22713804 PMC3522199

[12] Egan JF, Benn PA, Zelop CM, Bolnick A, Gianferrari E et al. (2004) Down syndrome births in the United States from 1989 to 2001. Am J Obstet Gynecol 191: 1044-1048. doi:10.1016/j.ajog.2004.06.050. PubMed: 15467587. 15467587

[13] Kajii T, Ferrier A, Niikawa N, Takahara H, Ohama K et al. (1980) Anatomic and chromosomal anomalies in 639 spontaneous abortuses. Hum Genet 55: 87-98. doi:10.1007/BF00329132. PubMed: 7450760. 7450760

[14] Snijders RJ, Sebire NJ, Nicolaides KH (1995) Maternal age and gestational age-specific risk for chromosomal defects. Fetal Diagn Ther 10: 356-367. doi:10.1159/000264259. PubMed: 8579773. 8579773

[15] Eiben B, Bartels I, Bähr-Porsch S, Borgmann S, Gatz G et al. (1990) Cytogenetic analysis of 750 spontaneous abortions with the direct-preparation method of chorionic villi and its implications for studying genetic causes of pregnancy wastage. Am J Hum Genet 47: 656-663. PubMed: 2220806. 2220806 PMC1683793

[16] Hsu JJ, Hsieh TT, Hsieh FJ (1996) Down syndrome screening in an Asian population using alpha-fetoprotein and free beta-hCG: a report of the Taiwan Down Syndrome Screening Group. Obstet Gynecol 87: 943-947. doi:10.1016/0029-7844(96)00042-7. PubMed: 8649703. 8649703

[17] Jou HJ, Shyu MK, Chen SM, Shih JC, Hsu JJ et al. (2000) Maternal serum screening for down syndrome by using alpha-fetoprotein and human chorionic gonadotropin in an asian population. a prospective study. Fetal Diagn Ther 15: 108-111. doi:10.1159/000020986. PubMed: 10720876. 10720876

[18] Hwa HL, Yen MF, Lin CL, Ko TM, Hsieh FJ et al. (2008) Cost-effectiveness analysis of triple test in second-trimester maternal serum screening for Down’s syndrome: an experience from Taiwan with decreasing birth rate but increasing population of old pregnant women. J Eval Clin Pract 14: 191-197. doi:10.1111/j.1365-2753.2007.00831.x. PubMed: 18284525. 18284525

[19] Shaw SW, Lin SY, Lin CH, Su YN, Cheng PJ et al. (2010) Second-trimester maternal serum quadruple test for Down syndrome screening: a Taiwanese population-based study. Taiwan. J Obstet Gynaecol 49: 30-34. 10.1016/S1028-4559(10)60005-8 20466289

[20] Gilbert RE, Augood C, Gupta R, Ades AE, Logan S et al. (2001) Screening for Down’s syndrome: effects, safety, and cost effectiveness of first and second trimester strategies. BMJ 323: 423-425. doi:10.1136/bmj.323.7310.423. PubMed: 11520837. 11520837 PMC37550

[21] Nicolaides KH (2001) Screening for fetal aneuploidies at 11 to 13 weeks. Prenat Diagn 31: 7-15. 10.1002/pd.2637 21210475

[22] Maiz N, Valencia C, Kagan KO, Wright D, Nicolaides KH (2009) Ductus venosus Doppler in screening for trisomies 21, 18 and 13 and Turner syndrome at 11-13 weeks of gestation. Ultrasound Obstet Gynecol 33: 512-517. doi:10.1002/uog.6330. PubMed: 19338027. 19338027

[23] MacRae AR, Chodirker BN, Davies GA, Palomaki GE, Knight GJ et al. (2010) Second and first trimester estimation of risk for Down syndrome: implementation and performance in the SAFER study. Prenat Diagn 30: 459-466. PubMed: 20440734. 20440734 10.1002/pd.2502

[24] Palomaki GE, Kloza EM, Lambert-Messerlian GM, Haddow JE, Neveux LM et al. (2011) DNA sequencing of maternal plasma to detect Down syndrome: an international clinical validation study. Genet Med 13: 913-920. doi:10.1097/GIM.0b013e3182368a0e. PubMed: 22005709. 22005709

[25] Chiu RW, Akolekar R, Zheng YW, Leung TY, Sun H et al. (2011) Non-invasive prenatal assessment of trisomy 21 by multiplexed maternal plasma DNA sequencing: large scale validity study. BMJ 342: c7401. doi:10.1136/bmj.c7401. PubMed: 21224326. 21224326 PMC3019239

[26] WHO (2003) World atlas for birth defects. 2nd edition. Geneva: World Health Organization.

[27] Olsen CL, Cross PK (1997) Trends in the use of prenatal diagnosis in New York State and the impact of biochemical screening on the detection of Down syndrome: 1984-1993. Prenat Diagn 17: 1113-1124. doi:10.1002/(SICI)1097-0223(199712)17:12. PubMed: 9467808. 9467808

[28] Khoshnood B, De Vigan C, Vodovar V, Goujard J, Goffinet F (2004) A population-based evaluation of the impact of antenatal screening for Down’s syndrome in France, 1981-2000. BJOG 111: 485-490. doi:10.1111/j.1471-0528.2004.00117.x. PubMed: 15104615. 15104615 PMC1894648

[29] Lai FM, Woo BH, Tan KH, Huang J, Lee ST et al. (2002) Birth prevalence of Down syndrome in Singapore from 1993 to 1998. Singapore Med J 43: 070-076. PubMed: 11993893. 11993893

