# Correction: The Impact of Down Syndrome Screening on Taiwanese Down Syndrome Births: A Nationwide Retrospective Study and a Screening Result from a Single Medical Centre

**DOI:** 10.1371/annotation/f6830f30-4b8e-4d72-a8c0-53ac7e50b440

**Published:** 2013-12-20

**Authors:** Shin-Yu Lin, Chia-Jung Hsieh, Yi-Li Chen, S. W. Steven Shaw, Ming-Wei Lin, Pau-Chung Chen, Chien-Nan Lee

There is an error in the name of the fourth author. The first name should be "S.W. Steven", and the last name should be "Shaw". The correct citation is:

Lin S-Y, Hsieh C-J, Chen Y-L, Shaw SWS, Lin M-W, et al. (2013) The Impact of Down Syndrome Screening on Taiwanese Down Syndrome Births: A Nationwide Retrospective Study and a Screening Result from a Single Medical Centre. PLoS ONE 8(9): e75428. doi:10.1371/journal.pone.0075428 

